# RAR‐Related Orphan Receptor Gamma (ROR‐γ) Mediates Epithelial‐Mesenchymal Transition Of Hepatocytes During Hepatic Fibrosis

**DOI:** 10.1002/jcb.25776

**Published:** 2017-04-27

**Authors:** Sung Min Kim, Jung Eun Choi, Wonhee Hur, Jung‐Hee Kim, Sung Woo Hong, Eun Byul Lee, Joon Ho Lee, Tian Zhu Li, Pil Soo Sung, Seung Kew Yoon

**Affiliations:** ^1^ The Catholic University Liver Research Center and WHO Collaborating Center of Viral HepatitisSeocho‐guSeoul06591Republic of Korea; ^2^ Molecular Medicine Research Center, School of Medical ScienceChifeng UniversityChifeng024000China; ^3^ Department of Internal Medicine, Seoul St. Mary's Hospital#505 Banpo‐Dong, Seocho‐gu, The Catholic University of KoreaSeoul06591Republic of Korea

**Keywords:** ROR‐γ, LIVER FIBROSIS, EPITHELIAL‐MESENCHYMAL TRANSITION, TRANSFORMING GROWTH FACTOR‐β1

## Abstract

The epithelial‐mesenchymal transition (EMT) is involved in many different types of cellular behavior, including liver fibrosis. In this report, we studied a novel function of RAR‐related orphan receptor gamma (ROR‐γ) in hepatocyte EMT during liver fibrosis. To induce EMT in vitro, primary hepatocytes and FL83B cells were treated with TGF‐β1. Expression of ROR‐γ was analyzed by Western blot in the fibrotic mouse livers and human livers with cirrhosis. To verify the role of ROR‐γ in hepatocyte EMT, we silenced ROR‐γ in FL83B cells using a lentiviral short hairpin RNA (shRNA) vector. The therapeutic effect of ROR‐γ silencing was investigated in a mouse model of TAA‐induced fibrosis by hydrodynamic injection of plasmids. ROR‐γ expression was elevated in hepatocyte cells treated with TGF‐β1, and ROR‐γ protein levels were elevated in the fibrotic mouse livers and human livers with cirrhosis. Knockdown of ROR‐γ resulted in the attenuation of TGF‐β1‐induced EMT in hepatocytes. Strikingly, ROR‐γ bound to ROR‐specific DNA response elements (ROREs) in the promoter region of TGF‐β type I receptor (Tgfbr1) and Smad2, resulting in the downregulation of Tgfbr1 and Smad2 after silencing of ROR‐γ. Therapeutic delivery of shRNA against ROR‐γ attenuated hepatocyte EMT and ameliorated liver fibrosis in a mouse model of TAA‐induced liver fibrosis. Overall, our results suggest that ROR‐γ regulates TGF‐β‐induced EMT in hepatocytes during liver fibrosis. We suggest that ROR‐γ may become a potential therapeutic target in treating liver fibrosis. J. Cell. Biochem. 118: 2026–2036, 2017. © 2016 The Authors. *Journal of Cellular Biochemistry* Published by Wiley Periodicals Inc.

Liver fibrosis is characterized by excessive accumulation of extracellular matrix proteins (ECM), leading to a distortion of the liver microstructure [Albanis and Friedman, 2001; Friedman, 2003, 2008]. Frequently, advanced liver fibrosis leads to liver cirrhosis, which is one of the major causes of mortality and liver transplantation worldwide [Albanis and Friedman, 2001]. It is well known that hepatic stellate cells (HSCs) are a major source of fibrogenic liver cells as they acquire unique features of the myofibroblasts responsible for the synthesis and deposition of ECM following chronic liver injury [Eng and Friedman, 2000; Bataller and Brenner, 2001].

Epithelial‐mesenchymal transition (EMT) is involved in many different types of cellular behavior, including embryonic development, tumor invasion, and tissue fibrosis [Thiery et al., 2009]. It is a dynamic cellular process that allows polarized immotile epithelial cells to lose their typical epithelial characteristics and become motile mesenchymal‐like cells [Kalluri and Neilson, 2003]. During EMT, epithelial markers, such as E‐cadherin, ZO‐1, and cytokeratin are downregulated, whereas mesenchymal markers, such as vimentin, fibronectin, and N‐cadherin, are upregulated [Kalluri and Weinberg, 2009].

Among the various cytokines and growth factors, TGF‐β has been implicated as a key factor in EMT in the liver [Bi et al., 2012]. TGF‐β is known to be a major pro‐fibrogenic cytokine that regulates cell proliferation, differentiation, apoptosis, and ECM expression [Dennler et al., 2002; Miyazawa et al., 2002]. It triggers EMT by binding heterodimeric type I and type II receptor complexes [Itoh et al., 2000; ten Dijke and Hill, 2004], leading to the activation of Smad‐dependent or Smad‐independent signaling. In the Smad‐dependent signaling pathway, phosphorylated Smad2, and Smad3 bind to Smad4 in the nucleus, and this trimetric complex induces expression of EMT regulatory factors, such as Snail, Slug, Twist, Zeb1, and Zeb2 [Cano et al., 2000; Bolos et al., 2003]. On the other hand, in Smad‐independent signaling, TGF‐β activates mitogen‐activated protein kinases (MAPKs), including ERK, JNK, and p38/MAP kinases, which induce the expression of EMT factors [Aomatsu et al., 2011].

TGF‐β1‐induced EMT is accompanied by the upregulation of E‐cadherin transcription repressors in primary murine hepatocytes [Kaimori et al., 2007; Nitta et al., 2008; Hernandez‐Gea and Friedman, 2011]. Zeisberg et al. [2007] reported that hepatocyte EMT was observed in CCl_4_‐induced liver fibrosis, and they also demonstrated that inhibition of the TGF‐β pathway limited the extent of liver fibrosis.

The RAR‐related orphan receptor (ROR) family consists of three members of the nuclear receptor family (ROR‐α, ROR‐β, and ROR‐γ, encoded by *RORA*, *RORB*, and *RORC*, respectively) that are known to have different physiological functions [Medvedev et al., 1996]. In particular, the RORC gene produces two different transcript variants. *RORC* transcript variant 2, which encodes one isoform called ROR‐γt, is expressed in T cells and is critical in the differentiation of naïve CD4+ T cells into T helper 17 cells (Th17 cells) [Eberl and Littman, 2003; Yang et al., 2008; Jetten, 2009]. These cells are involved in the pathogenesis of autoimmune disease, including psoriasis, rheumatoid arthritis, and nonalcoholic steatohepatitis (NASH) [Huang et al., 2007; Huh and Littman, 2012]. The other isoform, ROR‐γ, is different from ROR‐γt in the sequence of the N‐terminus and is expressed in many different tissues, including adipose tissue, muscle, the kidney, and the liver [Zhang et al., 2015]. In contrast to ROR‐γt, little is known about the function of ROR‐γ. Only a few studies have reported that ROR‐γ functions in the regulation of hepatic glucogenesis, insulin sensitivity, adipogenesis, and circadian expression of clock genes [Meissburger et al., 2011; Takeda et al., 2012, 2014].

In this study, we investigated the role of ROR‐γ in an in vitro EMT model using cultured primary hepatocytes and the anti‐fibrotic effect of ROR‐γ short‐hairpin RNA (shRNA) on thioacetamide (TAA)‐induced liver fibrosis in a mouse model. We identified ROR‐γ as a novel EMT‐regulating transcription factor in hepatocytes during hepatic fibrogenesis.

## MATERIALS AND METHODS

### ANIMAL STUDIES

Six‐week‐old BALB/C mice were used for the fibrosis induction experiments. They were divided into two groups, the carbon tetrachloride (CCl_4_; Sigma–Aldrich, St Louis, MO) and thioacetamide (TAA; Sigma) groups. For the CCl_4_‐injection group, the mice were injected intraperitoneally with CCl_4_ mixed with mineral oil (1:4) every 3 days for 8 weeks to induce liver fibrosis. For the TAA injection group, the mice were randomly divided into four groups: control, shROR‐γ‐injected (shROR‐γ, 30 µg/mouse via a hydrodynamic injection system), TAA‐treated (TAA, 100 mg/kg body weight by intraperitoneal injection) and shROR‐γ, and TAA co‐injected (TAA + shROR‐γ). TAA and ROR‐γ treatments were both given every 3 days for 8 weeks. The mice were sacrificed and their livers harvested. Isolation of primary hepatocytes, stellate cells (HSCs), endothelial cells (ECs), and Kupffer cells (KCs) from mouse liver was performed as previously described [Taura et al., 2008].

### CELL CULTURE

Non‐tumorigenic mouse hepatocyte cells, FL83B cells, were cultured in Ham's F‐12 K medium containing 10% FBS (Invitrogen, Carlsbad, CA), 100 µg/ml penicillin, and 0.25 µg/ml streptomycin and maintained at 37°C in a humidified incubator with 5% CO_2_. To induce EMT, the cells were treated with 5 ng/ml of TGF‐β1 for 48 h (R&D System, Minneapolis, MN).

### GENERATION OF ROR‐γ‐SILENCED CELL LINES

Transfection of siRNA targeting ROR‐γ (Sigma, SASI_Mm01_00068648) was performed using Lipofectamine 2000 (Invitrogen) following the manufacturer's protocol [Dalby et al., 2004].

To establish a stable ROR‐γ knockdown cell line, FL83B cells were infected with a mouse ROR‐γ specific shRNA encoded lentivirus (Sigma). Three coding regions targeting the mouse ROR‐γ starting positions 526 (LV‐shROR‐γ‐1), 1133 (LV‐shROR‐γ‐2), and 1597 (LV‐shROR‐γ‐3) in the sequence (GenBank Acc. No. NM 011281.1) were selected as shRNA target sequences. An shRNA negative control lentiviral particle (LV‐Control) was used as a negative control. To generate a stable cell line, FL83B cells were plated at a density of 1 × 10^5^ cells per 60‐mm culture dish and infected overnight with five multiplicity of infection (MOI) lentiviral particles in the presence of 8 µg/ml hexadimethrine bromide (Sigma). After infection, the transduced cells were selected using 10 µg/ml puromycin (Sigma) for 2 weeks and incubated at 37°C in a humidified incubator with 5% CO_2_. Suppression of ROR‐γ expression in selected cells was confirmed by Western blot analysis.

### QUANTITATIVE REAL‐TIME PCR

To detect the expression of mRNAs, complementary DNA (cDNA) was synthesized using RNA from isolated hepatocytes, HSCs, KCs, ECs, and ROR‐γ knockdown cells as previously described [Hur et al., 2012]. PCR was performed using specific primers (Supplementary Table S1), and Taqman probe‐based real‐time PCR amplifications were conducted with a Light Cycler 480 instrument (Roche Applied Science, Indianapolis, IN) in a total reaction volume of 20 µl.

### WESTERN BLOTTING, IMMUNOHISTOCHEMISTRY, AND IMMUNOFLUORESCENCE

Western blot analysis and Immunofluorescence staining were performed as previously described [Lee et al., 2014b]. Hematoxylin‐Eosin staining (H&E) and immunohistochemistry (IHC) were performed as previously described [Hur et al., 2010]. The stained slides were detected using an inverted microscope (Olympus, Tokyo, Japan) and confocal microscopy (Zeiss, Jena, Germany). All primary antibodies are listed in Supplementary Table S2.

### CHROMATIN IMMUNOPRECIPITATION

Genomic DNA prepared from LV‐shControl and LV‐shROR‐γ cells was crosslinked with 1% formaldehyde and fragmented into 0.5 kb segments by sonication. Soluble chromatin was then incubated overnight with an anti‐ROR‐γ antibody or control IgG. Finally, the immunoprecipitated DNA fragments were amplified and quantified by PCR using the following PCR primers for the *Smad2* promoter (5′‐AGTGGATTTCCAAGCCGATT ‐3′ and 5′‐GAAAAAGGGAATAGGGGGCA‐3′, the *Tgfbr1* promoter (5′‐CAGATCCTCTGGGAATGTGTA‐3′ and 5′‐TGCCCTTTCATCCATAGTTCT‐3′) and the *N‐cadherin* promoter (5′‐GGGTTGAAGCACTTACACTG ‐3′ and 5′‐AAATCAAACACCCACCACCT‐3′).

### MEASUREMENT OF LIVER COLLAGEN

Sirius Red staining was performed using the Picro–Sirius Red Staining Kit (Abcam, Cambridge, UK) to evaluate collagen deposition in liver tissues. Collagen deposition was also quantified using a hydroxyproline assay kit (BioVision, Milpitas, CA) according to the manufacturer's protocol.

### LIVER TISSUE OF PATIENTS WITH CHRONIC LIVER DISEASES

The study was approved in accordance with the ethical guidelines set forth by the Institutional Review Board of Seoul St. Mary's Hospital at the Catholic University of Korea (KC15TISI0605). A cohort of two groups, chronic hepatitis B and liver cirrhosis, was investigated. Each group comprised 14 liver biopsy tissues stored in liquid nitrogen. They were obtained from liver biopsies of patients with chronic live diseases prior to 2010. All patients provided written informed consents for the storage of liver tissue samples according to the ethical guidelines of Seoul St. Mary's Hospital in the Catholic University of Korea. Their personal information was restricted to analytical purposes. Such information is not available to the public.

### STATISTICAL ANALYSIS

All experiments were performed at least three times. Statistical comparisons were performed by *t*‐test using Sigma Plot (Systat Software, San Joes, CA). A *P*‐value <0.05 was considered significant.

## RESULTS

### INCREASED ROR‐γ PROTEIN LEVELS IN THE LIVERS OF PATIENTS WITH CIRRHOSIS

**Figure 1:**
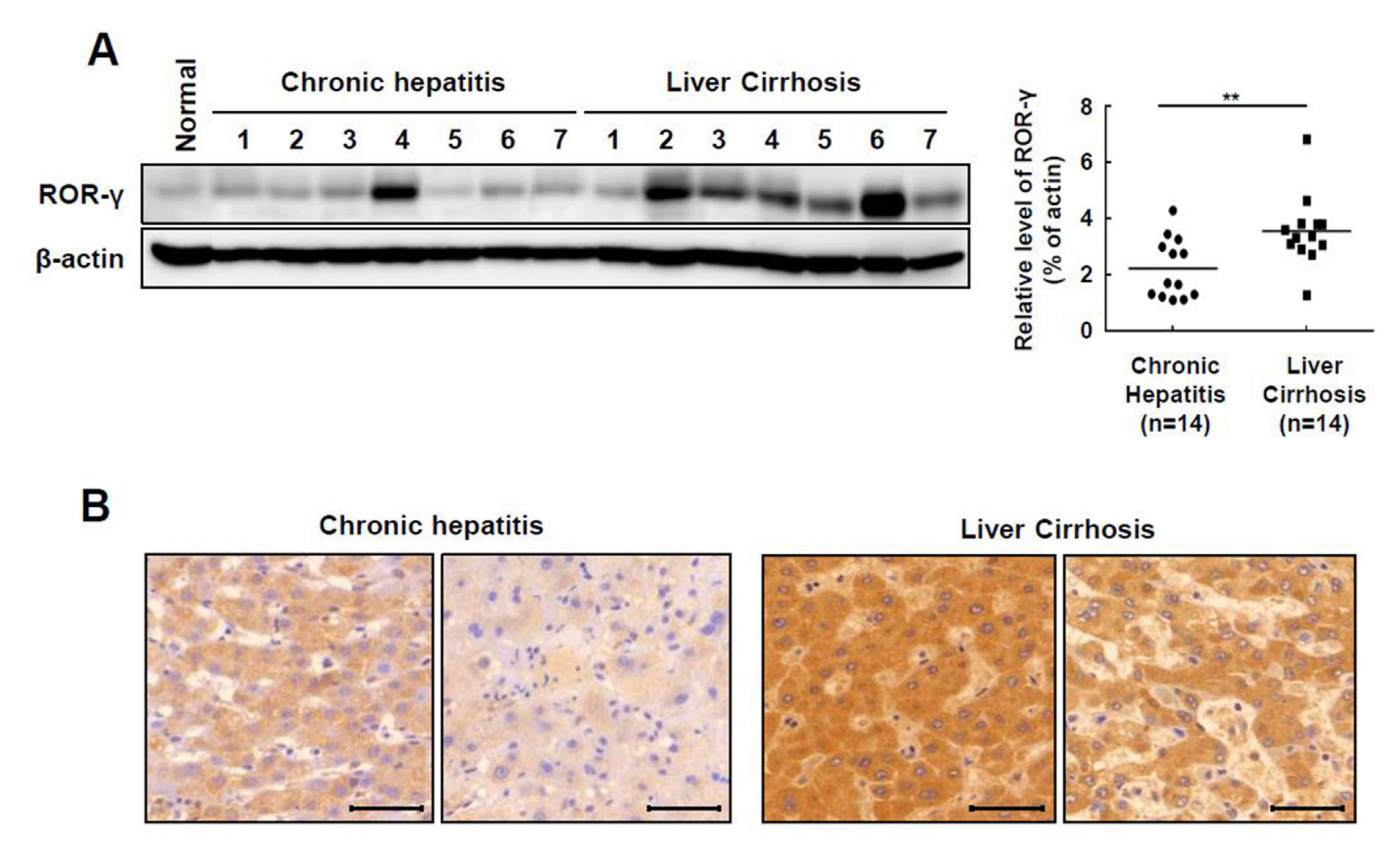
The ROR‐γ protein level is increased in the livers of patients with cirrhosis. (**A**) The expression of ROR‐γ was determined using Western blot in liver tissues with chronic hepatitis (n = 7) and cirrhosis (n = 7). The relative protein expression was normalized to β‐actin as a reference. (**B**) Immunohistochemistry was performed to determine the protein level of ROR‐γ in the livers of chronic hepatitis and cirrhosis patients. Original magnification: 400×. The values are reported as the mean ± standard error of the mean. All data are representative of at least three independent experiments. **P < 0.01.

First, we evaluated the ROR‐γ protein expression in clinical samples. Western blot analysis was conducted with the livers of patients with chronic hepatitis or cirrhosis. The expression of ROR‐γ was dramatically upregulated in livers of cirrhotic patients compared with livers of patients with chronic hepatitis (Fig. 1A). Immunohistochemical analysis showed ROR‐γ was more strongly expressed in the hepatocytes of cirrhotic livers than in the hepatocytes of livers with chronic hepatitis (Fig. 1B). Interestingly, ROR‐γ was expressed in both the nucleus and cytoplasm of the hepatocytes (Fig. 1B).

### UPREGULATION OF ROR‐γ IN HEPATOCYTES IN A MOUSE MODEL OF LIVER FIBROSIS

**Figure 2:**
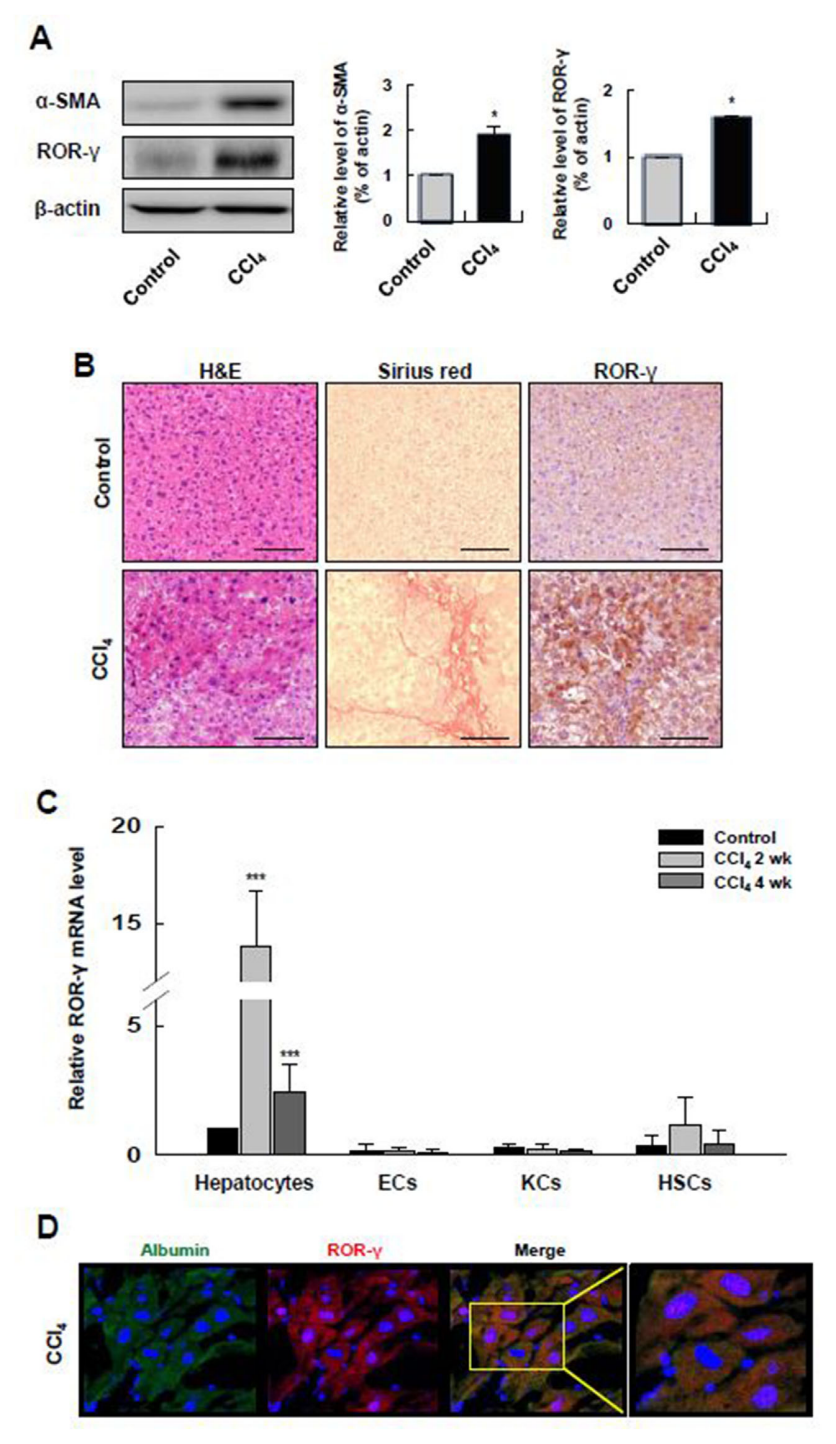
ROR‐γ is upregulated in the hepatocytes of a mouse model of liver fibrosis. (**A**) The protein levels of α‐SMA, ROR‐γ and β‐actin were determined in CCl_4_‐induced fibrotic liver tissues by Western blot. The relative expressions were normalized to β‐actin as a reference. The graph shows quantitative densitometry from the Western blot. (**B**) Histological analyses were performed on dissected fibrotic liver tissues after CCl_4_‐injection. The fibrotic liver tissues were stained with H&E, Sirius red, and antibodies against ROR‐γ. Original magnification: 100×. (**C**) ROR‐γ mRNA expression was evaluated by qRT‐PCR in different types of cells isolated from the liver of control and CCl_4_‐injected mice after 2 weeks or 4 weeks. (D) Double immunofluorescence staining for albumin (green) and ROR‐γ (red) performed in tissue samples control and CCl_4_‐injected mice. Nuclei were stained with DAPI (blue). Original magnification: 400×. Control: liver tissues of mock‐injected mice; CCl_4_ 2 wk: liver tissues of CCl_4_‐injected mice 2 weeks after injection; and CCl_4_ 4 wk: liver tissues from CCl_4_‐injected mice 4 weeks after injection. Values are presented as the mean ± standard error of the mean (n = 3). All data are representative of at least three independent experiments. *P < 0.05, **P < 0.01, ***P < 0.001.

Next, we examined ROR‐γ expression in a mouse model of CCl_4_‐induced liver fibrosis. Fibrosis was evaluated using the fibrotic marker α‐SMA and Sirius Red staining. The expression of ROR‐γ, as well as that of α‐SMA, increased in the CCl_4_‐injected mouse liver compared with the mock‐injected mouse liver (Fig. 2A). Sirius Red showed increased collagen in the CCl_4_‐injected mouse liver, and the expression of ROR‐γ was upregulated in this fibrotic liver (Fig. 2B). Consistent with the data presented in Figure 1B, the expression of ROR‐γ was more evident in the hepatocytes than in the other cell types (Fig. 2B). To determine which cell types within the liver contribute to the observed increase of ROR‐γ expression, we separated hepatocytes, KCs, ECs, and HSCs from the livers of CCl_4_‐injected and mock‐injected mice and analyzed the ROR‐γ mRNA expression in these four different cell types. Consistent with our immunohistochemical staining results, we found that ROR‐γ mRNA was mainly expressed in the hepatocytes during fibrogenesis. Notably, the ROR‐γ mRNA level was significantly increased in 2 weeks after CCl_4_‐injection, which is initial step of EMT to induce hepatic fibrogenesis. Therefore, it suggests that the increasing expression of ROR‐γ implicated EMT process during initiating hepatic fibrogenesis (Fig. 2C). These results were further confirmed by double immunofluorescence staining that showed co‐localization of the protein expression of ROR‐γ and albumin, a marker of hepatocytes (Fig. 2D).

### ROR‐γ IS UPREGULATED IN HEPATOCYTES AFTER IN VITRO TGF‐β1 STIMULATION

**Figure 3:**
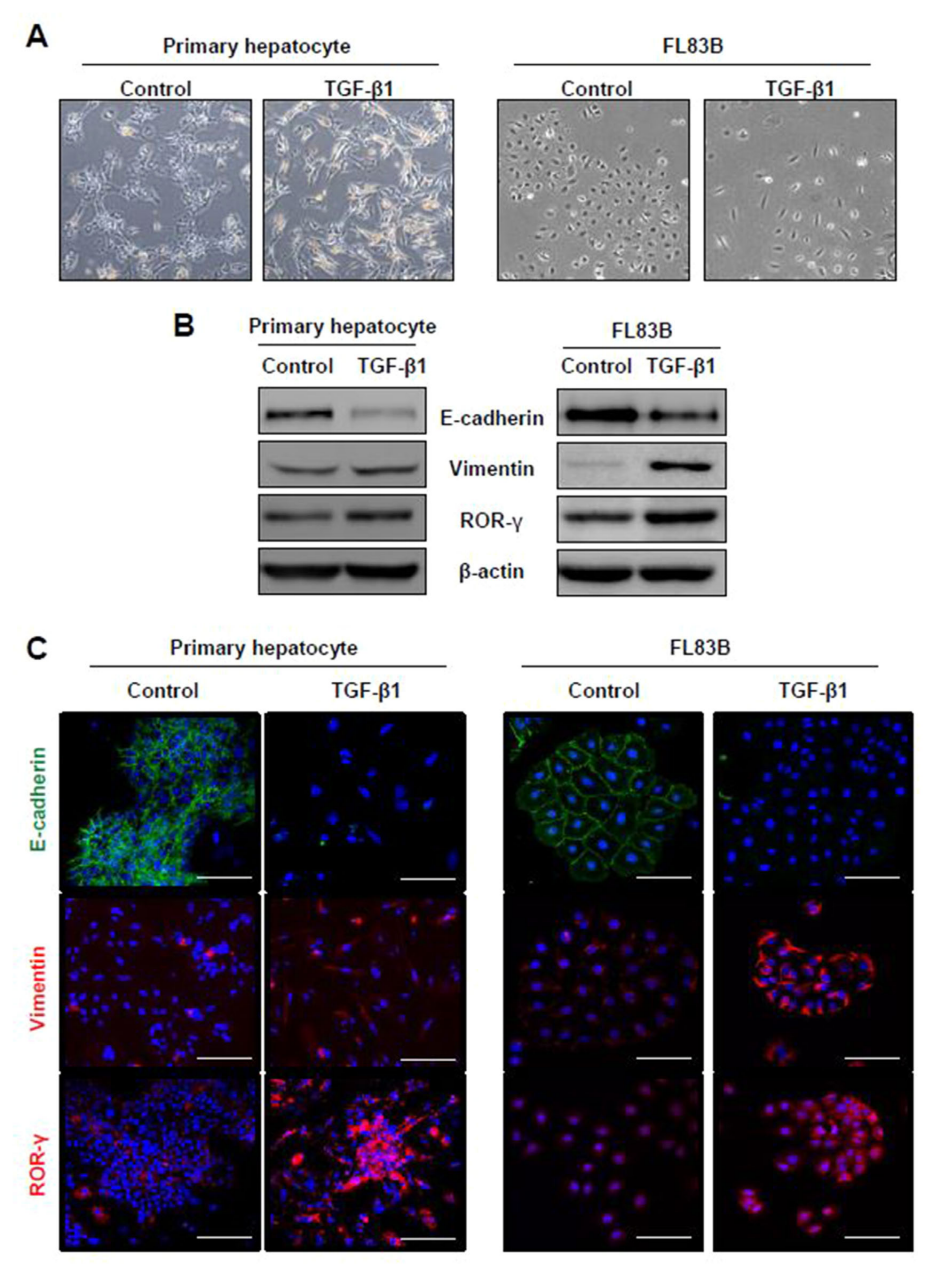
ROR‐γ is upregulated in hepatocytes after in vitro TGF‐β1 stimulation. (**A**) Using phase‐contrast imaging, morphological changes were observed in primary hepatocytes and FL83B cells treated with TGF‐β1 or left untreated. (**B**) Expression of EMT markers and ROR‐γ was determined by Western blot. The relative expression was normalized to β‐actin as a reference. (**C**) Immunofluorescence staining showed E‐cadherin (green), vimentin (red), and ROR‐γ (red) expression in TGF‐β1‐treated or untreated hepatocytes. Nuclei were stained with DAPI (blue). Original magnification: 200×. All data are representative of at least three independent experiments.

To investigate the role of ROR‐γ in hepatocytes during fibrogenesis, we established an in vitro EMT model by treating primary hepatocytes and a normal mouse hepatocyte cell line, FL83B, with TGF‐β1. After TGF‐β1 treatment, the shape of the primary hepatocytes and FL83B cells changed from an epithelial to a fibroblast‐like morphology (Fig. 3A). We found that the expression of E‐cadherin, a universal epithelial marker, decreased significantly and that the expression of vimentin was increased after TGF‐β1 treatment. In this setting, the expression of ROR‐γ increased significantly (Fig. 3B). Immunofluorescence staining showed a lack of E‐cadherin expression and the presence of vimentin filaments after TGF‐β1 treatment. Along with these changes, the ROR‐γ protein level was elevated in both the nucleus and the cytoplasm after TGF‐β1 treatment (Fig. 3C). These results demonstrate that ROR‐γ upregulation may be associated with EMT.

### SILENCING ROR‐γ CAUSES UPREGULATION OF EPITHELIAL MARKERS

**Figure 4:**
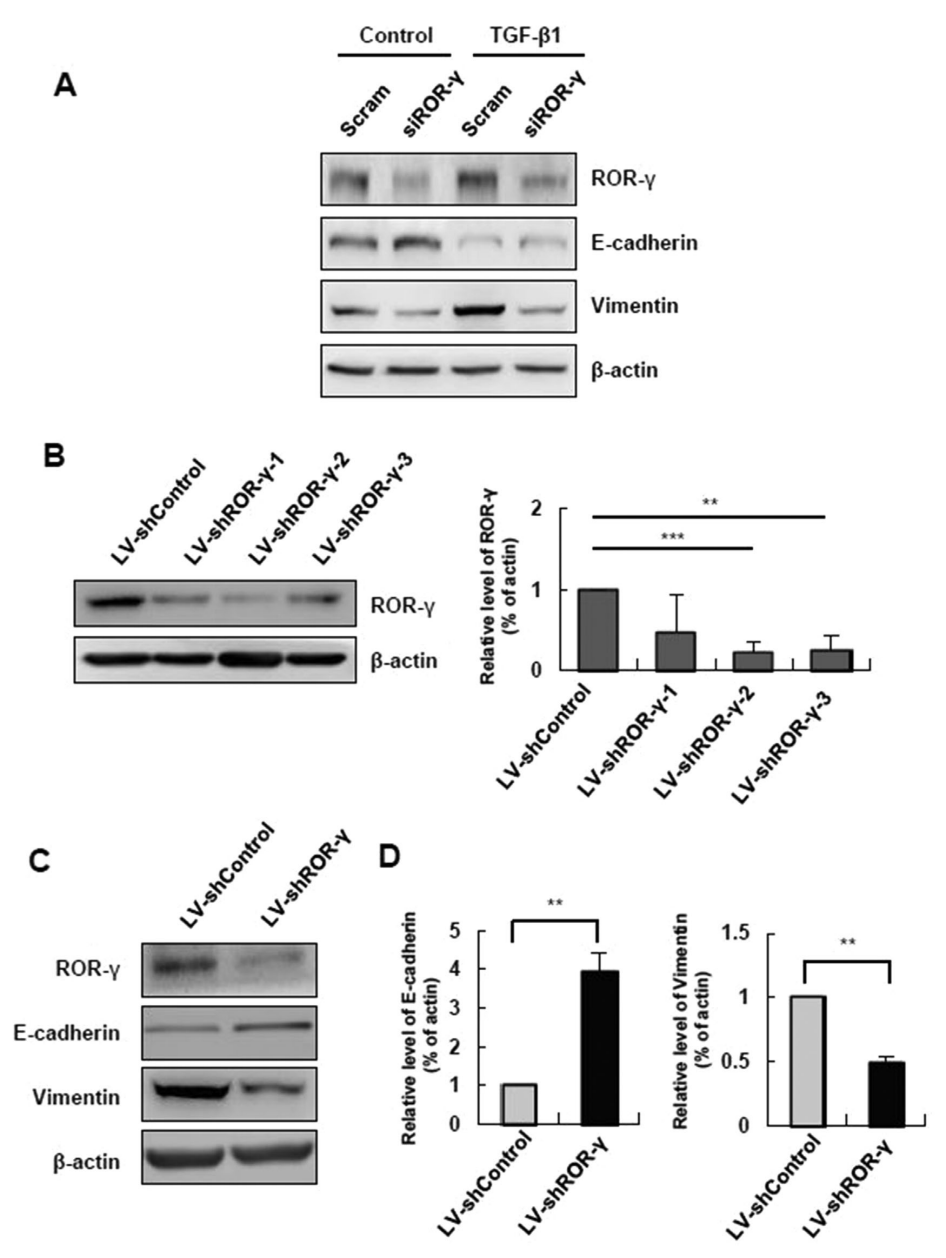
Silencing of ROR‐γ causes upregulation of epithelial markers. (**A**) ROR‐γ, E‐cadherin and vimentin protein levels were determined by Western blot in hepatocytes treated with TGF‐β1 and scrambled siRNA and siROR‐γ or untreated hepatocytes. The relative expression was normalized to β‐actin as a reference. (**B**) To evaluate ROR‐γ knockdown efficacy, the expression level of ROR‐γ was determined after transduction of FL83B with various types of LV‐shROR‐γ. The relative expression was normalized to β‐actin as a reference. (**C**) Expression of E‐cadherin and vimentin was determined by Western blot. The relative expressions were normalized to β‐actin expression as a reference. Scram: scrambled siRNA‐treated cells; siROR‐γ: siRNA targeting ROR‐γ‐targeting cells; LV‐shControl: non‐targeting shRNA‐encoding lentivirus; LV‐shROR‐γ‐1‐3: ROR‐γ shRNA‐encoding lentivirus. All data are representative of at least three independent experiments *P < 0.05, **P < 0.01, ***P < 0.001.

To assess whether the TGF‐β‐induced EMT process is influenced by ROR‐γ silencing, FL83B cells were transfected with siROR‐γ or scrambled siRNA, followed by TGF‐β1 treatment. After ROR‐γ siRNA transfection, E‐cadherin expression was restored, and vimentin expression was decreased (Fig. 4A). These results suggest that ROR‐γ expression in hepatocytes plays a pivotal role in TGF‐β‐induced EMT.

To suppress ROR‐γ expression effectively, we established a stable ROR‐γ knockdown cell line using a shRNA lentivirus infection system. First, we assessed three different sequences of shRNA targeting ROR‐γ. Among the cells expressing shRNA that targeted ROR‐γ, LV‐shROR‐γ‐2 cells exhibited the most efficient silencing of ROR‐γ expression compared with LV‐Control cells. Thus, we decided to use LV‐shROR‐γ‐2 cells for further analysis (Fig. 4B). As a result of the ROR‐γ knockdown, E‐cadherin was upregulated, and vimentin downregulated in this cell line (Fig. 4C). Taken together, these results demonstrated that silencing ROR‐γ may restore epithelial features to hepatocytes with epithelial properties.

### ROR‐γ REGULATES EMT THROUGH INDUCTION OF Tgfbr1 and Smad2

**Figure 5:**
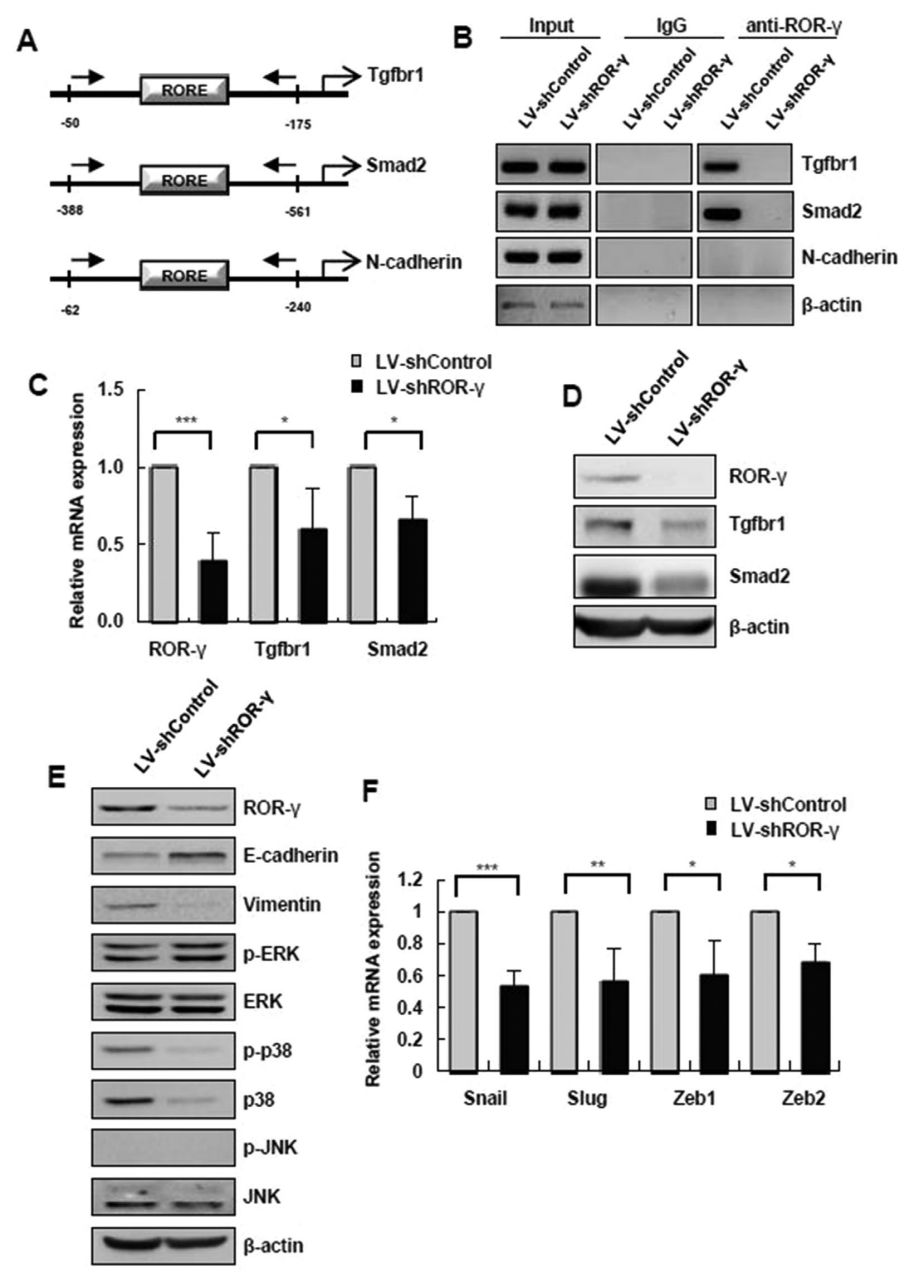
ROR‐γ regulates EMT through induction of Tgfbr1 and Smad2. (**A**) Schematic diagram of the primer design for the ChIP assay on the Smad2, Tgfbr1 and N‐cadherin promoter regions. (**B**) Results of the ChIP assay performed using anti‐ROR‐γ on the mouse Smad2, Tgfbr1 and N‐cadherin promoter in control or ROR‐γ‐silenced FL83B cells. ChIP primer regions are shown in (**A**). (**C**) mRNA levels of Tgfbr1 and Smad2 were measured by qRT‐PCR. (**D**) Expression of Tgfbr1, Smad2 and β‐actin was determined by Western blot. The relative expression was normalized to β‐actin expression as a reference. (**E**) Molecules involved in the Smad‐independent pathway were detected by Western blot analysis. **(F**) The mRNA levels of EMT regulatory factors were evaluated by qRT‐PCR. LV‐shControl: non‐targeting shRNA‐encoding lentivirus; LV‐shROR‐γ‐1‐3: ROR‐γ shRNA‐encoding lentivirus. The values are presented as the mean ± standard error of the mean. All data are representative of at least three independent experiments. *P < 0.05, ***P < 0.001.

ROR‐γ is known to bind as a monomer to a specific sequence called “RORE,” consisting of an AGGTCA “half‐site” with a 5′ AT‐rich extension [Medvedev et al., 1996; Jetten et al., 2001]. In order to elucidate the mechanism by which ROR‐γ is involved in hepatocyte EMT, we looked for ROREs in the promoters of several genes. We found three putative targets, Smad2, Tgfbr1, and N‐cadherin, from the previously reported microarray data of Mukherji et al. [2013] (Fig. 5A). A ChIP assay showed that the bands corresponding to the ROR‐γ binding promoter region of Smad2 or Tgfbr1 appeared in LV‐Control cells, whereas the bands had disappeared in ROR‐γ‐knockdown cells (Fig. 5B). These results confirm that Tgfbr1 and Smad2 are direct target genes of ROR‐γ in hepatocytes. We then confirmed Tgfbr1 and Smad2 expression in ROR‐γ‐silenced cell. In line with these results, the expression of Tgfbr1 and Smad2 at both the mRNA (Fig. 5C) and protein levels (Fig. 5D) decreased significantly after ROR‐γ knockdown. To determine the influence of Smad‐independent signaling through Tgfbr1 in EMT, we further analyzed the expression of ERK, p38, and JNK MAP kinases in ROR‐γ‐knockdown cells. Interestingly, p38 was downregulated, while the expression of ERK and JNK were unchanged (Fig. 5E). It is well known that Smad‐dependent and ‐independent signaling initiate transcription of EMT regulatory genes, such as Snail, Slug, Zeb1, and Zeb2, following activation by TGF‐β1. Snail, Slug, Zeb1, and Zeb2 were significantly downregulated at the mRNA level when the ROR‐γ expression was silenced (Fig. 5F). Collectively, these results show that ROR‐γ specifically enhances Tgfbr1 and Smad2 expression, resulting in upregulation of Snail, Slug, Zeb1, and Zeb2 expression via Smad‐dependent and ‐independent pathways.

### ROR‐γ SILENCING AMELIORATES THE PATHOLOGICAL CHANGES TO THE LIVER IN AN ANIMAL MODEL OF TAA‐INDUCED LIVER FIBROSIS

**Figure 6:**
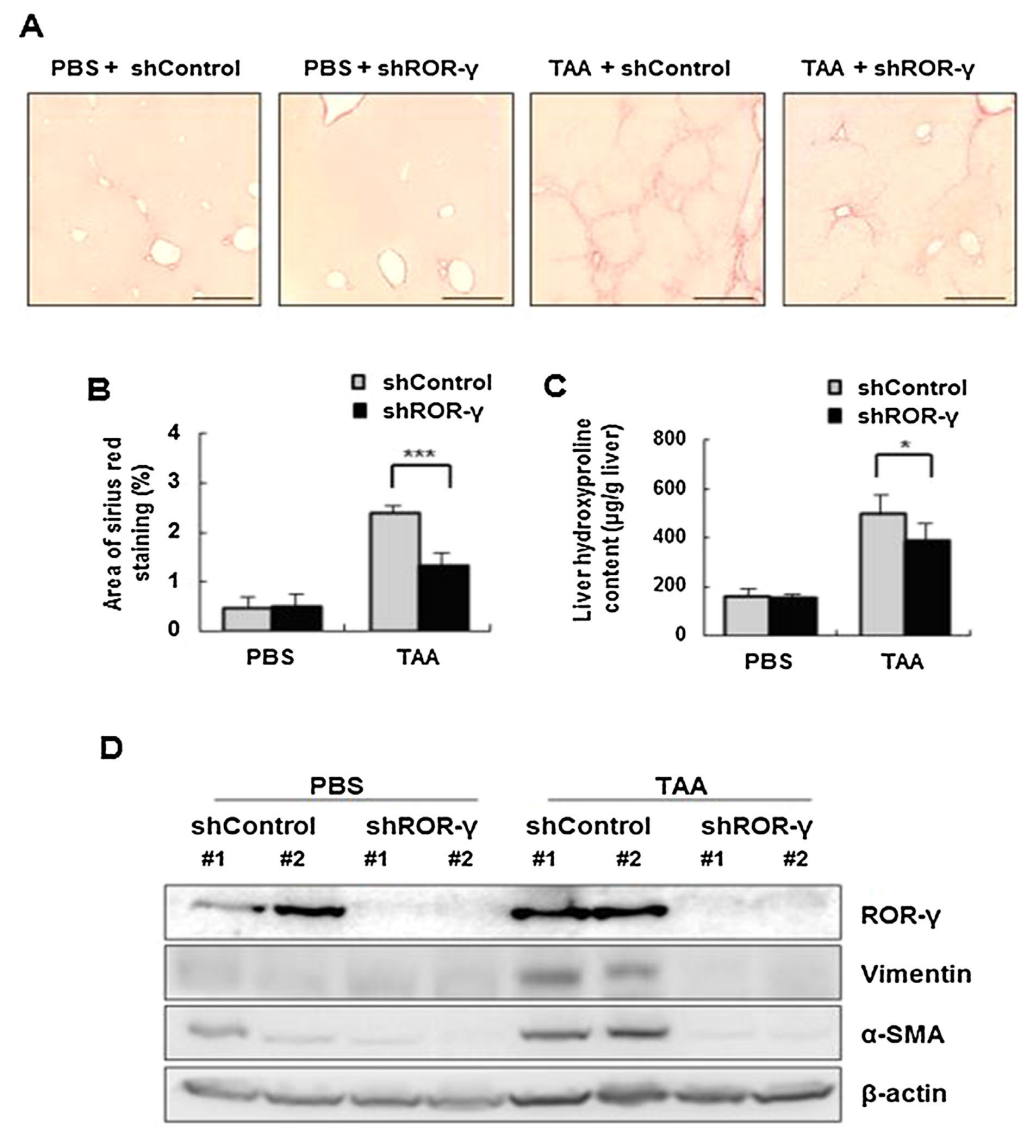
ROR‐γ silencing ameliorates the pathological changes of the liver in the animal model of TAA‐induced liver fibrosis. Mice were intraperitoneally injected with 100 mg/kg TAA three times a week. Hydrodynamic injection of 30 µg of shControl or shROR‐γ plasmids were performed through the tail vein for 8 weeks. (**A** and **B**) Morphometric analysis was performed on Sirius Red‐stained liver sections (n = 5 fields/liver) to measure collagen deposition. Original magnification: 100×. (**C**) The amount of liver hydroxyproline was detected. (**D**) Expression of ROR‐γ, vimentin, α‐SMA and β‐actin was determined by Western blot. The relative expression was normalized to β‐actin expression as a reference. The values are presented as the mean ± standard error of the mean. PBS, phosphate‐buffered saline (PBS; TAA, Thiacetamide (TAA), shControl: non‐targeting shRNA‐encoding plasmid; shROR‐γ, ROR‐γ shRNA‐encoding plasmid). All data are representative of at least three animals. *P < 0.05, ***P < 0.001.

Finally, we verified the role of ROR‐γ in vivo in an animal model of liver fibrosis. Following acute TAA liver injury, severe fibrosis was observed that was similar to the above results obtained from CCl_4_‐injection (Fig. 6A). However, the severity of fibrosis in shROR‐γ‐injected, TAA‐treated mice was substantially lower than that of the shControl‐injected, TAA‐treated mice (Fig. 6A) in terms of the area of fibrosis (%) (Fig. 6B) and the amount of fibrous tissue, measured by hepatic hydroxyproline content (Fig. 6C). Moreover, administration of TAA showed upregulation of vimentin and α‐SMA, which was not observed in the shROR‐γ‐injected TAA‐treated group (Fig. 6D). These results demonstrate that in vivo silencing of ROR‐γ may ameliorate the pathological changes to the liver in an animal model of TAA‐induced liver fibrosis.

## DISCUSSION

The aim of the present study was to investigate the role of ROR‐γ during liver fibrosis in relation to hepatocyte EMT. First, we investigated the expression of ROR‐γ in human fibrotic liver disease samples. We found that ROR‐γ was dramatically upregulated in human livers with cirrhosis. Interestingly, the upregulation was limited to the hepatocytes of the cirrhotic livers. Although the expression of ROR‐γ started to increase just after liver injury, the expression peaked 2 weeks after CCl_4_‐injection, in parallel with the activity of intrahepatic TGF‐β [Zeisberg et al., 2007; Rowe et al., 2011]. This implies that the function of ROR‐γ is tightly linked with TGF‐β1‐dependent hepatic fibrogenesis. Next, we examined the expression of ROR‐γ in the animal models of CCl_4_‐ and TAA‐induced liver fibrosis. Both animal models have been widely used as experimental tools of chronic liver injury followed by hepatic fibrosis without damage to other organs and may mimic the condition of human chronic liver disease [Wang et al., 2012; Lee et al., 2014a]. We observed pathological abnormalities and collagen deposition after intraperitoneal injection of CCl_4_ and TAA. At the same time, the expression of ROR‐γ and the fibrosis marker α‐SMA were increased in the hepatocytes of damaged livers. Then, we investigated the effects of silencing ROR‐γ using the TAA‐induced animal model. Silencing ROR‐γ ameliorated the pathologic changes of mouse livers and decreased collagen deposition.

To identify the mechanisms by which ROR‐γ works in the hepatocytes during fibrosis, we focused EMT. In 2007, Zeisberg et al. [2007] and Kaimori et al. [2007] reported that hepatocytes play a substantial role in liver fibrosis through EMT. However, another report showed that hepatocytes did not have a collagen‐expressing phenotype in mice chronically treated with CCl_4_ [Taura et al., 2010]. Although it is premature to conclude whether EMT actually occurs and contributes to fibrogenesis by collagen production during human liver injury, it seems clear that mesenchymal markers are upregulated in hepatocytes by the TGF‐β produced during liver injury and fibrosis. To date, previous reports showing that Snail deletion in hepatocytes attenuates liver fibrosis [Rowe et al., 2011; Nieto et al., 2016] have also suggested that hepatocyte EMT may play a role in liver fibrosis. Accordingly, EMT‐regulating genes, such as Snail1 [Rowe et al., 2011] and HNF4α [Yue et al., 2010], have been emerging as targets as part of a new concept for treating liver fibrosis. Among these EMT‐regulating genes, transcription factors have emerged as important regulators of the EMT process. Importantly, ROR‐γ is a DNA‐binding transcription factor that binds to a specific sequence called “RORE” to transcribe target genes [Jetten et al., 2001]. Mukherji et al. [2013] published microarray data for ROR‐γ target genes that contain the RORE sequence in their promoter region. Based on these microarray data, we demonstrated for the first time that ROR‐γ can directly bind to Tgfbr1 and Smad2 in hepatocytes. Tgfbr1 and Smad2 are very important in the TGF‐β pathway during EMT. The TGF‐β1 ligand preferentially signals from the TGF‐β type II receptor (Tgfbr2) to the Tgfbr1 to activate the Smad‐dependent or Smad‐independent signaling pathway [Derynck and Zhang, 2003]. Our data show that silencing ROR‐γ not only downregulated Tgfbr1 and Smad2 but also decreased p38 MAPK expression in ROR‐γ‐knockdown cells. However, the mechanisms by which ROR‐γ decreases p38 MAPK expression still remain to be elucidated. We can only infer that ROR‐γ may indirectly regulate p38 MAPK expression. Accordingly, our data showed that ROR‐γ can regulate EMT‐promoting factors, such as Snail, Slug, Zeb1, and Zeb2, through transcription of Tgfbr1 and Smad2. Collectively, we have identified ROR‐γ as a novel EMT‐regulating transcription factor that exerts its effects by binding to the RORE‐containing promoter regions of Tgfbr1 and Smad2.

Overall, our data show that ROR‐γ regulates hepatocyte EMT via Smad‐dependent or Smad‐independent signaling pathways. Further, ROR‐γ is upregulated in human cirrhotic livers and fibrosis‐induced mouse livers, and in vivo silencing ROR‐γ attenuated EMT and ameliorated the liver pathology. We suggest that ROR‐γ may become a potential therapeutic target in treating liver fibrosis.

## Supporting information

Additional supporting information may be found in the online version of this article at the publisher's web‐site.

**Table S1**. Primers used for PCR and ChIP assay.
**Table S2**. Primary antibodies used for immunodetection.
