# Supplementary material for: RAR‐Related Orphan Receptor Gamma (ROR‐γ) Mediates Epithelial‐Mesenchymal Transition Of Hepatocytes During Hepatic Fibrosis
Source: J Cell Biochem. 2017 Apr 27;118(8):2026–36. doi: 10.1002/jcb.25776 (PMC5488206; doi:10.1002/jcb.25776)
Supplement: Supplementary file 1 — Table S1. Primers used for PCR and ChIP assay. Table S2. Primary antibodies used for immunodetection. [file JCB-118-2026-s001.docx]

**Supplementary data to:**

**RAR-related orphan receptor gamma (ROR-γ) mediates epithelial-mesenchymal transition of hepatocytes during hepatic fibrosis**

Sung Min Kim^1^, Jung Eun Choi^1^, Wonhee Hur^1^, Jung-Hee Kim^1^, Sung Woo Hong^1^, Eun Byul Lee^1^, Joon Ho Lee^1^, Tian zhu Li^3^, Pil Soo Sung^1,2^ and Seung Kew Yoon^1,2^

**Table of Contents**

**Supplementary Table S1**………………...…………………………………………………2

**Supplementary Table S2**………………...…………………………………………………3

**Supplementary Table S1.** Primers used for PCR and ChIP assay

| **Gene** | **Species** | **Forward primer** | **Reverse primer** |
| --- | --- | --- | --- |
| Rorγ | mouse | TGA GGA TGA GAT TGC CCT CTA | TTG CAG ATG TTC CAC TCT CCT |
| Tgfbr1 | mouse | TGG AGC AAC ATG TGG AAC TC | GTC AGC AGC CGG TTA CCA |
| Smad2 | mouse | AGG ACG GTT AGA TGA GCT TGA G | GTC CCC AAA TTT CAG AGC AA |
| Snail1 | mouse | CTT GTG TCT GCA CGA CCT GT | CAGGAGAATGGCTTCTCACC |
| Slug | mouse | CAG TGC AAT TTA TGC AAT AAG ACC | CAG TGC AGC TGC TTG TGT TT |
| Zeb1 | mouse | ACT GCC AGC AGA CCA GAC A | TCA CAC TCG TTG TCT TTC ACG |
| Zeb2 | mouse | TGC GTC CAC TAC GTT GTC AT | CAAGAGGCGCAAACAAGC |
| Smad2 (RORE) | mouse | AGT GGA TTT CCA AGC CGA TT | GAA AAA GGG AAT AGG GGG CA |
| TGFBR1 (RORE) | mouse | CAG ATC CTC TGG GAA TGT GTA | TGCCCTTTCATCCATAGTTCT |
| N-cadherin (RORE) | mouse | GGG TTG AAG CAC TTA CAC TG | AAA TCA AAC ACC CAC CAC CT |

**Supplementary Table S2.** Primary antibodies used for immunodetection.

| **Name** | **Manufacturer** | **catalog number** | **Species** | **Dilution** |
| --- | --- | --- | --- | --- |
| E-cadherin  Vimentin  ROR-γ  Smad2/3  Tgfbr1  ERK  p-ERK  p38/MAPK  p-p38/MAPK  JNK  p-JNK  α-SMA  β-actin  FITC-albumin | Cell Signaling  Cell Signaling  Abcam  Cell Signaling  Abcam  Santa Cruz  Cell Signaling  Cell Signaling  Cell Signaling  Santa Cruz  Cell Signaling  Sigma-Aldrich  Sigma-Aldrich  Bethyl Labs | 3195S  57741S  Ab78007  8685p  ab31013  sc-93  #9101  8690p  9211s  SC-137018  #9251  A2547  A1978  #A90-234F | R  R  R  R  R  R  R  R  R  M  R  M  M  M | 1:1000 (WB) 1:100 (IF)  1:1000 (WB) 1:100 (IF)  1:500 (WB) 1:250 (IHC) 1:100 (IF)  1:1000 (WB)  1:500 (WB)  1:1000 (WB)  1:1000 (WB)  1:1000 (WB)  1:500 (WB)  1:1000 (WB)  1:500 (WB)  1:500 (WB)  1:2000 (WB)  1:200 (IF) |

M, mouse; R, rabbit; WB, western blot; IF, immunofluorescence; IHC, immunohistochemistry.
